# A New Strategy to Control and Eradicate “Undruggable” Oncogenic K-RAS-Driven Pancreatic Cancer: Molecular Insights and Core Principles Learned from Developmental and Evolutionary Biology

**DOI:** 10.3390/cancers10050142

**Published:** 2018-05-14

**Authors:** Robert E. Van Sciver, Michael P. Lee, Caroline Dasom Lee, Alex C. Lafever, Elizaveta Svyatova, Kevin Kanda, Amber L. Collier, Lauren L. Siewertsz van Reesema, Angela M. Tang-Tan, Vasilena Zheleva, Monicah N. Bwayi, Minglei Bian, Rebecca L. Schmidt, Lynn M. Matrisian, Gloria M. Petersen, Amy H. Tang

**Affiliations:** 1 Department of Microbiology and Molecular Cell Biology, Leroy T. Canoles Jr. Cancer Research Center, Eastern Virginia Medical School, Norfolk, VA 23507, USA; VanSciRE@EVMS.EDU (R.E.V.S.); SvyatoE@EVMS.EDU (E.S.); KandaK@EVMS.EDU (K.K.); Monicah.Bwayi@STJUDE.ORG (M.N.B.); bianm@yahoo.com (M.B.); 2 School of Medicine, Eastern Virginia Medical School, Norfolk, VA 23507, USA; LeeMP@EVMS.EDU (M.P.L.); LeeDC@EVMS.EDU (C.D.L.); LafeveAC@EVMS.EDU (A.C.L.); CollieAL@EVMS.EDU (A.L.C.); SiewerLL@EVMS.EDU (L.L.S.v.R.); 3 Department of Molecular and Cell Biology, University of California at Berkeley, Berkeley, CA 94720, USA; angelatangtan@berkeley.edu; 4 Department of Surgery, Eastern Virginia Medical School, Norfolk, VA 23507, USA; vasilena.zheleva@gmail.com; 5 Department of Biology, Upper Iowa University, Fayette, IA 52142, USA; Schmidtr@uiu.edu; 6 Pancreatic Cancer Action Network, 1500 Rosecrans Ave, Suite 200, Manhattan Beach, CA 90266, USA; lmatrisian@pancan.org; 7 Department of Health Sciences Research, Mayo Clinic Cancer Center, Mayo Clinic Pancreatic Cancer SPORE, BioBusiness 5-85, 200 First Street SW, Rochester, MN 55905, USA; Petersen.gloria@mayo.edu

**Keywords:** oncogenic K-RAS mutations, oncogenic K-RAS pathway activation, SINA and SIAH family of RING domain E3 ligases, evolutionary conservation, ubiquitin-mediated proteolysis, signaling gatekeeper, pancreatic tumor vulnerability, oncogenic K-RAS-driven tumor relapse and metastasis

## Abstract

Oncogenic K-RAS mutations are found in virtually all pancreatic cancers, making K-RAS one of the most targeted oncoproteins for drug development in cancer therapies. Despite intense research efforts over the past three decades, oncogenic K-RAS has remained largely “undruggable”. Rather than targeting an upstream component of the RAS signaling pathway (i.e., EGFR/HER2) and/or the midstream effector kinases (i.e., RAF/MEK/ERK/PI3K/mTOR), we propose an alternative strategy to control oncogenic K-RAS signal by targeting its most downstream signaling module, Seven-In-Absentia Homolog (SIAH). SIAH E3 ligase controls the signal output of oncogenic K-RAS hyperactivation that drives unchecked cell proliferation, uncontrolled tumor growth, and rapid cancer cell dissemination in human pancreatic cancer. Therefore, SIAH is an ideal therapeutic target as it is an extraordinarily conserved downstream signaling gatekeeper indispensable for proper RAS signaling. Guided by molecular insights and core principles obtained from developmental and evolutionary biology, we propose an anti-SIAH-centered anti-K-RAS strategy as a logical and alternative anticancer strategy to dampen uncontrolled K-RAS hyperactivation and halt tumor growth and metastasis in pancreatic cancer. The clinical utility of developing SIAH as both a tumor-specific and therapy-responsive biomarker, as well as a viable anti-K-RAS drug target, is logically simple and conceptually innovative. SIAH clearly constitutes a major tumor vulnerability and K-RAS signaling bottleneck in pancreatic ductal adenocarcinoma (PDAC). Given the high degree of evolutionary conservation in the K-RAS/SIAH signaling pathway, an anti-SIAH-based anti-PDAC therapy will synergize with covalent K-RAS inhibitors and direct K-RAS targeted initiatives to control and eradicate pancreatic cancer in the future.

## 1. Introduction

Pancreatic cancer represents the archetype of metastatic cancers with a dismal five-year survival rate of 9% and a median survival of 6 months post-diagnosis [1,2,3]. It is currently the third leading cause of cancer-related death, despite only constituting roughly 3% of estimated new cancer cases each year. Its mortality rate exceeded that of breast cancer in 2017, and it is projected to surpass colorectal cancer to become the second leading cause of cancer-related death in the United States by 2030 [1,4]. In 2018 alone, an estimated 55,440 patients will be newly diagnosed with pancreatic cancer, and 44,330 patients are expected to die from this deadly malignancy [4]. Clinical symptoms associated with pancreatic ductal adenocarcinoma (PDAC) are exacerbated by late diagnosis, local invasion, and systemic metastases, culminating in poor prognosis and high mortality [3,5,6]. Currently, surgical resection offers the best hope for long-term survival, but only in cases where the disease is discovered early, prior to local and distant metastases [2,7,8,9]. Only one-fifth (~20%) of pancreatic cancer patients are resectable, while the remainder (~80%) of patients are found to have inoperable disease at the time of diagnosis [1,10,11]. Of patients with operable disease, surgical resection is a curative measure for only 20% of those diagnosed with early stage and non-metastatic diseases. They ultimately represent a tiny subset (4–5%) of all pancreatic cancer patients who become long-term survivors. The remaining 80% of those with operable disease experience post-operative tumor relapse and eventually succumb to this deadly disease [10,12,13,14,15,16]. For the vast majority of patients with inoperable disease, chemo- and radiation therapies are offered as palliative care treatments, yielding few long-term survivors [17,18,19]. The clinical reality is that there are no reliable biomarkers for early detection, and the multi-modality, high-intensity chemo- and radiation therapies confer only a modest survival benefit to patients with chemo-resistant, relapsed, and malignant pancreatic cancer [20,21,22]. Thus, new and innovative strategies are urgently needed to reverse tumorigenesis, prevent metastasis, improve clinical outcomes, and increase overall survival of pancreatic cancer.

Recent advances in multi-agent chemotherapies (FOLFIRINOX or nab–paclitaxel–gemcitabine), neoadjuvant chemotherapies, and precision surgical techniques have improved perioperative morbidity and mortality in pancreatic cancer [2,9,20,22,23,24,25,26,27]. FOLFIRINOX is an aggressive chemo-regimen composed of four chemotherapy agents [folinic acid (leucovorin), fluorouracil (5-FU), irinotecan and oxaliplatin], and it is used to treat locally advanced and metastatic pancreatic cancer in young and physically fit patients [20,22]. FOLFIRINOX conferred a better median overall survival (OS) of 11.1 months versus 6.8 months, progression free survival (PFS) of 6.4 months versus 3.3 months, and an increased response rate of 31.6% versus 9.4% when compared to single-agent gemcitabine chemotherapy; however, significant toxicity and adverse effects to FOLFIRINOX were noted [20]. Moreover, a dual-agent chemotherapy of gemcitabine plus albumin-bound paclitaxel (nab-paclitaxel) was approved as a first line combination chemotherapy for patients with advanced and metastatic pancreatic cancer and conferred an improved median OS of 8.5 months versus 6.7 months, PFS of 5.5 months versus 3.7 months, and an increased response rate of 23% versus 7%, when compared to gemcitabine mono-therapy [22]. Recently, the concept of treating more pancreatic cancer patients with neoadjuvant chemotherapies prior to surgical resection has gained widespread support as a means to convert borderline unresectable pancreatic cancer to resectable status [25,28,29,30]. Despite these significant advances, the overall five-year survival rate for pancreatic cancer remains at 9%. This is the lowest among all solid human tumors, highlighting the unmet clinical need, intellectual challenge, and limited arsenal of effective therapies when it comes to controlling chemo-resistant, recurrent, and metastatic pancreatic cancer [1,2,4,31,32,33]. The clinical reality is that most patients with infiltrating PDAC will develop tumor relapse and succumb to this lethal disease, as metastatic pancreatic cancer remains incurable in the clinic [2,6,27,34,35,36]. As such, there is a pressing need to discover novel anti-PDAC strategies and identify sensitive and reliable molecular tools for early detection. It is imperative to develop therapy-responsive and predictive biomarkers for stratifying patients, quantifying tumor response, forecasting tumor relapse, and predicting patient survival. Importantly, state-of-the-art in vivo tumor tracking technologies and new breakthrough therapies are needed to guide individualized treatment modalities, exploit major PDAC tumor vulnerabilities, identify actionable targets, deliver precision medicine, and ultimately control and eradicate late-stage PDAC in the future.

PDAC tumors have a high degree of tumor and tumor microenvironment (TME) heterogeneity, signaling network plasticity, and genetic diversity [6,33,37]. Their rapidly evolving tumor genomes become far more pronounced in resistant, recurrent, and metastatic settings [38,39,40,41,42]. PDAC is known to have dense stroma with increased mechanical stiffness, hypovascularity, and poor perfusion leading to a hypoxic, nutrient-deprived, and immunosuppressive TME, altered autophagy and metabolic profiles, mitochondrial dysfunction, and heightened cellular stress pathways [3,43,44,45,46,47]. These genetic, biochemical, biophysical, and physiological features make PDAC tumors quite challenging for drug delivery and effective tumor targeting. PDAC is known to be an immunologically cold tumor with few tumor-specific neo-antigens, a lack of infiltrating immune cells, and poor antigenicity, failing to elicit robust immune responses to the existing immune checkpoint therapies. As such, CTLA-4 and/or PD-1/PD-L1 blockade, and stromal-targeted therapies have yet to show adequate efficacy against pancreatic cancer in the clinic [48,49,50,51,52,53,54]. Despite these impenetrable obstacles, there are several innovative strategies that aim to re-establish the responsiveness to checkpoint blockade therapies and redirect the host immune system to attack PDAC cells [55,56,57,58,59,60,61,62,63,64]. The question of how to precisely target and eradicate chemo-refractory, high-grade, and metastatic PDAC tumors has challenged our collective scientific wisdom and presents a barrier to improving standard of care (SOC) therapies, advancing personalized medicine, and inventing new lifesaving strategies to save more PDAC patients in the clinic. As such, it is imperative to design innovative and combination therapies by exploring key PDAC tumor vulnerabilities, deploying precision medicine tools, and shutting down the major tumor-driving K-RAS signaling pathway in order to achieve a curative measure in the future [1,2,6,35,65].

## 2. As a Central Tumor-Driving Signaling Pathway, Oncogenic K-RAS Activation Remains Largely “Undruggable” in Human Cancer Despite Three Decades of Intense Investigation

**Figure 1:**
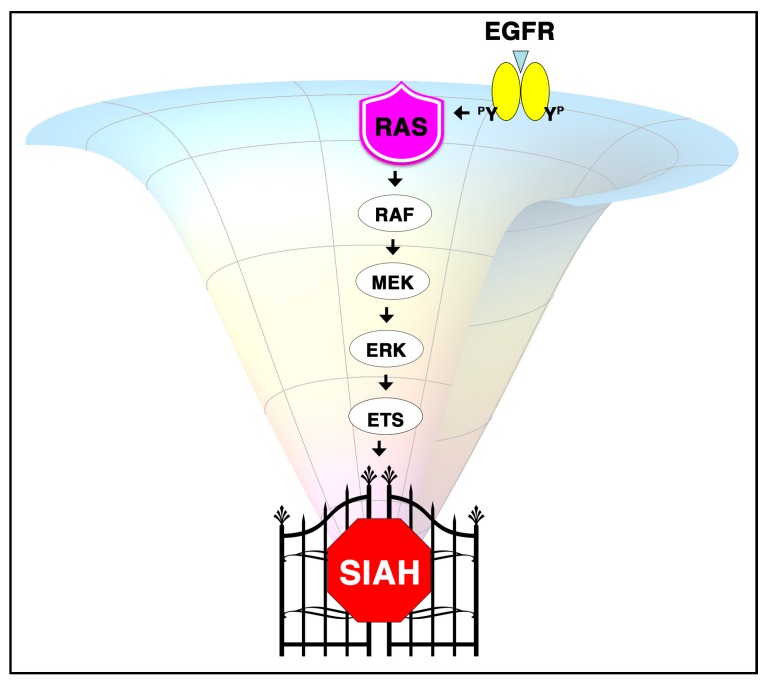
SIAH is the most downstream signaling gatekeeper and a key signaling bottleneck in the RAS signaling pathway. This schematic illustration highlights the critical role of SIAH, a key signaling bottleneck and the most downstream signaling gatekeeper, whose function is indispensable for proper RAS signal transduction. Oncogenic K-RAS pathway activation is known to exhibit immense signaling plasticity, heterogeneity, complexity, and spatio–temporal context- dependence in vitro and in vivo. A systemic view of the multifaceted RAS signaling network activation is depicted as an elastic signaling “funnel” that is highly flexible, adaptive, and constantly changing, and rapidly evolving. This signaling funnel depicts the dynamic regulation of a multitude of compensatory pathway activation, cross talk, signaling bifurcation, feed-forward and/or feedback control mechanisms that are commonly observed in response to oncogenic K-RAS pathway activation in a cancer cell signaling network. As active RAS signals propagate down the EGFR/RAS/RAF/MEK/ERK signal transduction cascade, SIAH constitutes the most downstream signaling hub. Its critical biological function is indispensable for controlling the active EGFR/RAS/RAF/MEK/ERK/ETS signaling output and readout in normal development as well as in K-RAS-driven oncogenesis. Supported by key insights and molecular principles gleaned from developmental, evolutionary and cancer biology, it is not surprising to find that mammalian SIAH serves as an important signaling gatekeeper and critical signaling bottleneck in the oncogenic K-RAS signaling pathway in human cancers. As such, SIAH is a major PDAC tumor vulnerability that is ideally positioned to become a next-generation viable and logical anti-K-RAS drug target in oncogenic K-RAS-driven human cancers.

Genomic landscape studies have indicated that oncogenic K-RAS pathway activation is virtually universal in pancreatic cancer [12,27,66,67,68,69]. As a major tumor-promoting pathway, the central importance of oncogenic K-RAS activation has been well established in human pancreatic cancer [5,12,35,67,70,71]. An important goal in pancreatic cancer biology is to identify a means to countervail hyperactive K-RAS signals and reverse malignant transformation. The current SOC therapies are rarely curative in resistant, relapsed, and metastatic settings. Therefore, there is a pressing need to identify early detection biomarkers, sensitive screening tools, lifesaving interventions, and innovative strategies to halt tumor growth and inhibit metastasis, and ultimately, to design better precision therapies that will improve overall patient survival in pancreatic cancer [2,3,5,6,12,35]. Pancreatic cancer, with its rapidly evolving tumor genomes, oncogenic K-RAS signaling plasticity and network adaptability, and immense tumor/TME heterogeneity, can easily bypass, rewire, and re-activate its formidable K-RAS signaling network via numerous possible combinations of synergistic compensatory RAS effector pathway activation to re-stimulate tumor growth. In its quest for tumor regrowth and full malignancy, PDAC develops multifaceted resistance mechanisms by overcoming several of the systemic signaling blockades induced by the current SOC therapies. Therefore, most of the existing anti-K-RAS-centered targeted therapies have failed and/or showed limited efficacy [37,68,69,70]. Based on the molecular insights and mechanistic principles learned from developmental, evolutionary, and cancer biology, we recognize that the value of revisiting the K-RAS core operational principles and redesigning an alternative K-RAS targeting program are of paramount importance in pancreatic cancer [72,73]. Hence, we propose to target the evolutionarily conserved and the most downstream signaling module, Seven-In-Absentia Homolog (SIAH), which functions as the ultimate signaling “gatekeeper” and an indispensable major oncogenic K-RAS vulnerability in PDAC. By using an alternative approach of choking its critical signaling bottleneck at the most downstream signaling hub, we aim to shut down oncogenic K-RAS hyperactivation, inhibit PDAC regrowth, prevent pancreatic cancer cell dissemination, and impede its systemic spread in vitro and in vivo (Figure 1).

RAS proteins (H-RAS, K-RAS, and N-RAS) are small GTPases that function as molecular switches. Conformational changes between active GTP-bound and inactive GDP-bound states, transduce signals from the upstream receptors, EGFR/HER2/RTK to midstream kinases to orchestrate a symphony of coordinated cell signaling events to control cell proliferation, differentiation, survival, and apoptosis in all multicellular organisms [66,70,74,75,76,77]. Most oncogenic K-RAS mutations compromise its ability to hydrolyze GTP, essentially retaining the aberrant K-RAS protein in the constitutively active GTP-bound state [69,78,79,80]. These constitutively active K-RAS oncoproteins are the most common mutations detected in pancreatic cancer [67,70,77,81,82]. Due to its unique enzyme kinetics and ultra-low Kd in the picomolar (pM) range, it is kinetically impossible to design competitive small molecule inhibitors to shut down K-RAS oncoproteins [78,79,80]. As such, oncogenic K-RAS has remained undruggable for the past three decades [37,69,83].

Hyperactivation of the oncogenic K-RAS signaling pathway endows PDAC tumor cells with synergistic compensatory pathway co-activation, increased RAS signaling network plasticity, and genomic and systemic adaptability, culminating in aggressive tumor growth, resistance to cytotoxic agents, systemic metastasis, and poor clinical outcomes [6,37,69,71,84]. Even in the absence of oncogenic RAS mutations, activation of the RAS signaling pathway constitutes the “major engine” that drives therapy resistance, aggressive tumor growth, relapse and metastasis in many other types of deadly human cancers [70,83,85,86]. Thus, RAS pathway activation is critical not only to promote tumor initiation, escape, and progression, but also to stimulate tumor relapse, therapy resistance, and metastatic dissemination of human cancer cells [87,88,89,90,91].

## 3. Targeting and Shutting Down Oncogenic K-RAS Hyperactivation Is the “Holy Grail” in Cancer Biology and Cancer Therapy

Oncogenic K-RAS activation is a well-known drug target in pancreatic cancer [72,78,79,82]. Many innovative approaches have brought great excitement into the RAS field. Several groundbreaking studies that directly target K-RAS exchange mutants and K-RAS membrane localization are a key part of the intense efforts to design specific, effective and durable anti-K-RAS inhibitors that shut down active K-RAS directly. Firstly, RAS must be lipid-modified and membrane-tethered for its biological activity. As such, farnesyltransferase inhibitors (FTI) have been pursued as potential anti-RAS based anticancer targets; however, these FTI-based therapies failed to demonstrate sufficient clinical efficacy, due to compensatory lipid modification moieties for proper K-RAS plasma membrane anchoring [92,93,94,95,96,97]. Recently, a new phosphodiesterase-δ (PDEδ) inhibitor, Deltazinone 1, binds to the prenyl-binding pocket of PDEδ, interferes with proper K-RAS-PDEδ interaction, impairs the proper plasma membrane attachment of prenylated K-RAS, and impedes proper K-RAS signaling in pancreatic cancer cells in vitro and in vivo [98,99,100]. Secondly, significant efforts have been made to inhibit the RAS signal transduction cascades at its upstream receptor tyrosine kinase (RTK) and midstream kinases. Unfortunately, multiple strategies to inhibit oncogenic K-RAS activation at its major effector pathways, the RAF/MEK/MAPK and/or PI3K/mTOR signaling pathways, have shown limited efficacy and transient effects, with rapid drug resistance developing clinically [2]. Thirdly, there are several exciting pharmacologic breakthroughs that have the potential to make oncogenic K-RAS a druggable target in human cancer [101]. These covalent K-RAS inhibitors can directly bind to the catalytic active site of K-RAS^G12C^ via a trapping mechanism, prevent the GTP/GDP exchange in its nucleotide-binding pocket, inactivate this constitutively active form of K-RAS^G12C^ oncoprotein, and disrupt K-RAS^G12C^ signaling to its downstream effector pathways [102,103,104,105,106,107,108,109]. A recent study reported that a highly selective K-RAS^G12C^-specific covalent inhibitor (ARS-1620) has shown promising potency in inducing K-RAS^G12C^-driven PDAC tumor regression in a patient-derived xenograft (PDX) mouse model of pancreatic cancer [109]. While PDAC tumor volumes were significantly reduced in response to the daily administration of ARS-1620 by oral gavage, it is unclear whether a durable and sustained tumor regression can be achieved, tumor recurrence can be prevented, or compensatory signaling pathway activation can be suppressed in response to this potent K-RAS^G12C^-specific inhibitor (ARS-1620) in the clinic [109]. It is important to note that the prevalence of this K-RAS^G12C^ mutation is uncommon (present in approximately 3% of PDACs), thus limiting the overall utility of deploying this specific covalent K-RAS^G12C^ inhibitor to improve patient survival in general [69,110,111,112]. Thus, improving the in vivo efficacy and duration of more K-RAS allele-specific covalent inhibitors is still necessary in order to unlock their clinical utility as potent anti-PDAC targeted agents [80]. Fourthly, since 2013, the National Institutes of Health (NIH) has established a new RAS initiative that streamlines and supports several highly competitive and innovative projects at the National Cancer Institute (NCI) that aim to directly target and shut down oncogenic K-RAS hyperactivation in preclinical and clinical settings. If successful, it will expedite the concerted efforts to shut down oncogenic K-RAS-driven tumors using the next-generation of covalent K-RAS inhibitors to benefit pancreatic cancer patients in the future [79,113]. Fifthly, at the Pancreatic Cancer Action Network (PanCAN), the state-of-the-art “Know Your Tumor” initiative has recommended molecular profiling of human pancreatic tumors to determine the best treatment options and to deliver the most promising results clinically. The combined national efforts at PanCAN and NCI are well-poised to unleash the synergistic power of applying genomic, genetic, epigenetic, transcriptomic, miRNA, proteomic, and kinomic approaches to human pancreatic cancer by identifying new tumor vulnerabilities and actionable targets, and delivering precision medicine to augment the existing treatment paradigms [27,34,36]. As such, the field of pancreatic cancer is rapidly moving forward, with many exciting results forthcoming at NCI, PanCAN, and many other academic institutions worldwide.

## 4. The Challenges of Targeting K-RAS-Driven Malignant Pancreatic Tumors

Oncogenic K-RAS hyperactivation successfully co-opts and corrupts PDAC tumor cells, and organizes their neighboring cells (fibroblasts, endothelial cells, and infiltrating immune cells) to form a resilient ecosystem and highly adaptive cellular community in the TME [37]. As such, targeting the K-RAS pathway is expected to be extremely difficult from a systems biology point of view. The first conceptual challenge is that oncogenic K-RAS signaling is nonlinear, highly dynamic, context-dependent, flexible, and constantly evolving. Secondly, oncogenic K-RAS activation is known to orchestrate co-activation of several compensatory effector pathways, stimulate signaling bifurcation and cross talk, induce systemic signaling adaptation via feed-forward and feedback mechanisms, synergize cancer network rewiring, and increase tumor cell survival in response to aggressive chemo- and radiation therapies. Thirdly, pancreatic cancer has immense genetic/epigenetic diversity, and rapidly evolving intra- and inter-tumor and TME heterogeneity in chemo-refractory and malignant tumors [38,39,41,42,84,114]. Fourthly, the rapid tumor genome evolution and high tumor/TME heterogeneity create an unpredictable, hypovascular, hypoxic, and desmoplastic cellular environment that is constantly changing as pancreatic cancer cells grow, disseminate, and metastasize. Therefore, it is conceptually challenging, if not impossible, to identify an “ideal” anti-K-RAS target and develop a precise and curative anti-PDAC therapy using these descriptive “-omics” assessments of malignant pancreatic tumors [2,33,40]. As such, oncogenic K-RAS remains “undruggable” despite significant efforts over the last three decades [37,69,78,79,83]. By revisiting the tenets of developmental and evolutionary biology, we propose an alternative approach by concentrating on the most evolutionarily conserved key signaling gatekeeper as well as the major signaling hubs of the RAS signaling pathway in hopes of further identifying major tumor vulnerabilities and actionable targets in pancreatic cancer.

## 5. Developmental Biology and Evolutionary Biology Are the Guiding Light in Cancer Biology

Many fundamental principles gleaned from simple model organisms have allowed us to identify and focus on evolutionarily conserved genes, signaling pathways, and regulatory mechanisms. Understanding these molecular basics is necessary to formulate conceptual breakthroughs, develop state-of-the-art investigative tools, and translate groundbreaking innovations from basic science to clinical research [115,116,117,118]. Historically, the core knowledge and fundamental principles acquired in model organisms have formed the major pillars of scientific investigations in modern cancer biology [119,120,121,122]. Among all the cancer signaling pathways that were initially described in model organisms, the RAS signaling pathway is one of the most studied and pertinent pathways to cancer biology. The RAS signaling pathway was discovered in *Drosophila melanogaster* and *Caenorhabditis elegans* [123,124,125]. It constitutes one of the best cases where key signaling molecules, regulatory mechanisms, compensatory pathways, and molecular interactions were revealed via extensive genetic epistasis studies in model organisms [123,124,125]. These studies established the core hierarchy of the RAS signal transduction cascade, revealed its critical downstream signaling gatekeepers, and demonstrated the fundamental RAS operational principles that are highly conserved across metazoan species. Several major RAS signaling hubs identified from model organisms are evolutionarily conserved, and thus strategically well positioned to serve as novel anti-RAS drug targets in the future. By targeting these critically important signaling hubs, a new evidence-based anti-RAS strategy, which is supported by developmental and evolutionary biology, has started to emerge. Developmental and evolutionary biology are the guiding light in cancer biology. It is conceivable that a key signaling hub-centered anti-K-RAS strategy will shut down oncogenic K-RAS activation and block oncogenic K-RAS-driven tumor growth and metastasis [73]. We therefore propose revisiting the fundamental biology of RAS activation, as well as RAS signaling plasticity, RAS network rewiring and escape mechanisms. Taking advantage of these molecular insights, core operational principles, evolutionarily conserved key signaling modules, and indispensable major signaling hubs of oncogenic RAS signaling will allow us to uncover important tumor vulnerabilities and identify actionable targets to control and conquer chemo-refractory, relapsed, and metastatic PDAC in the future.

## 6. Evidence from Developmental Biology and Evolutionary Biology in Support of Cancer Biology

### 6.1. SIAH Is an Extraordinarily Conserved Signaling Module and the Most Downstream Signaling “Gatekeeper” Indispensable for Proper RAS Signal Transduction in Metazoan Species

Genetic epistasis is a commonly used genetic technique to order gene mutations and organize gene interactions into a particular signaling pathway in *Drosophila* [126,127]. More than two decades ago, Dr. Gerald M. Rubin and his team at the University of California at Berkeley and the Howard Hughes Medical Institute (HHMI), were the first to (1) discover that RAS acts downstream of a receptor tyrosine kinase (RTK); (2) establish the genetic hierarchical order of the RAS signal transduction cascade; and (3) identify rat sarcoma viral oncogene (RAS), Sevenless (SEV), Seven-In-Absentia (SINA), and Son of Sevenless (SOS) through an elegant genetic screen [125,128,129,130,131,132,133]. The discovery constituted a major breakthrough in the RAS signaling field because the RAS small GTPase physically interacts with active RTK only transiently. As such, RAS could only be identified as a downstream signaling component in the RTK pathway via an elegant and powerful genetic screen [131]. By conducting multiple saturated genetic screens focusing on each signaling component of the RAS signaling pathway, Dr. Rubin and his team successfully established the detailed genetic epistasis of the key signaling molecules in the RAS signaling pathway. They sorted these genes into the upstream, mid-stream and downstream signaling components of the RAS pathway by using *Drosophila* eye development as a robust and sensitive readout system assaying for RAS activation or inactivation [125]. Through these meticulous genetic epistatic analyses of all the RAS signaling components identified thus far, SINA was identified as the most downstream signaling component essential for proper SEV/RAS/RAF/MEK/ERK/ETS signal transmission [125,130,132]. The RAS signaling pathway is highly conserved and its mode of operation in fruit flies is directly pertinent to the mammalian RAS signal transduction cascade.

Hyperactive K-RAS signaling is a major menace that drives aggressive tumor growth and cancer dissemination in human pancreatic cancer. Guided by these fundamental insights and molecular principles learned from developmental biology and evolutionary biology, we lay out an alternative strategy to control oncogenic K-RAS hyperactivation and eradicate oncogenic K-RAS-driven tumors in pancreatic cancer [72]. Instead of targeting the upstream and midstream signaling modules, we propose to stop malignant tumor growth of pancreatic cancer cells by targeting the most downstream signal module–the SIAH E3 ligases–in the oncogenic K-RAS signaling pathway [72,134,135] (Figure 1). We showed that SIAH deficiency impedes oncogenic K-RAS signaling transduction as well as oncogenic K-RAS-driven human pancreatic and lung cancer cells in xenograft mouse models [134,135]. Recently, we performed a phylogenetic analysis on the SIAH E3 ligases to show that SIAH is an extraordinarily conserved signaling component in the RAS signaling pathway in metazoa [73].

### 6.2. SINA-Mediated Proteolysis Is the Most Downstream Signaling Gatekeeper in the RAS Pathway in Drosophila Eye Development

**Figure 2:**
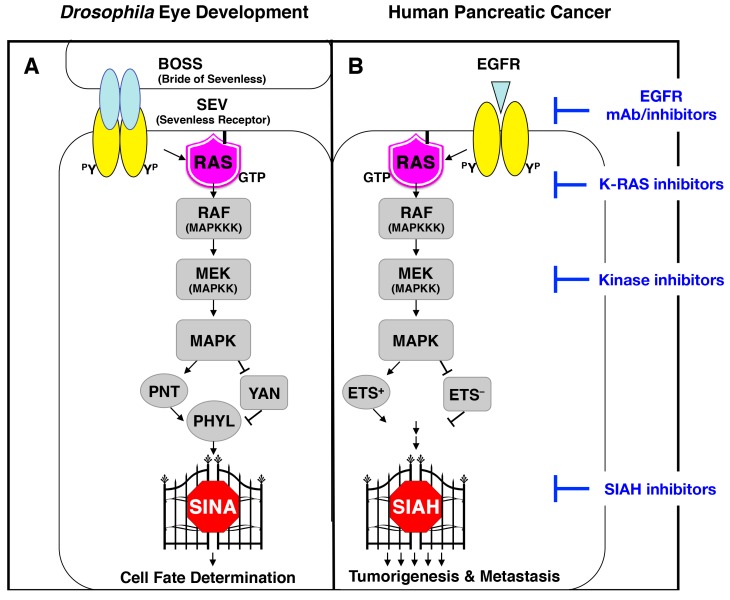
SIAH-dependent proteolysis is essential for K-RAS signal transduction in human pancreatic cancer. This schematic diagram shows that the RAS signaling pathway is highly conserved from fruit flies to humans. (**A**) A schematic illustration of the *Drosophila* RAS signal transduction pathway is shown. SINA is the most downstream signaling gatekeeper identified in the *Drosophila* RAS pathway, and its function is required for the proper transmission of active EGFR/RAS/RAF/MEK/MAPK/ETS signal in vivo; (**B**) A schematic illustration of the human RAS signal transduction pathway is shown. The human RAS pathway has an identical signaling cascade and the same key downstream signaling modules as discovered in *Drosophila*. The extensive evolutionary conservation observed in the RAS-SIAH signaling axis suggests that the gatekeeper function of SINA/SIAH is likely to be conserved from invertebrates to vertebrate animals. Therefore, analogous to *Drosophila* SINA, inhibiting human SIAH function may provide a novel and innovative means to inhibit oncogenic K-RAS-mediated tumorigenesis and metastasis in pancreatic cancer.

The RAS signal transduction cascade is one of the most evolutionarily conserved signaling pathways in the animal kingdom [77,83,136,137,138,139]. It is a critically important signaling pathway that controls many fundamental cellular processes, including cell proliferation, differentiation, migration, apoptosis, and stem cell renewal during normal development, tissue regeneration, and pathogenesis of diseases [70,77,123,125,140]. The simple model organism, *Drosophila melanogaster*, has played a pivotal role in identifying many key signaling components and delineating the regulatory principles of the RAS signal transmission, including Sevenless (SEV, the *Drosophila* homolog of mammalian EGFR), Son of Sevenless (SOS), rat sarcoma viral oncogene (RAS), RAF serine/threonine kinase, Mitogen-activated Protein Kinase Kinase (MEK), Mitogen-activated Protein Kinase (MAPK), and Seven-In-Absentia (SINA) [125,128,130,131,141,142,143,144]. SINA is a ubiquitin E3 ligase whose function is absolutely required for RAS signal transduction and R7 photoreceptor cell fate determination in *Drosophila* eye development (Figure 2A) [125,128,145]. Extensive genetic epistatic studies have demonstrated that SINA activity is indispensable for proper RAS signal transduction, and that none of the active EGFR, RAS, RAF, and MAPK signals can be transmitted without proper SINA biological activity [125,128,130,145]. Thus, *Drosophila* SINA is the most downstream signaling “gatekeeper” identified in the RAS signaling pathway in vivo [72,125,128,145]. These genetic studies have revealed key insights and core principles about the critical dependence of SINA activity for proper RAS signal transduction in *Drosophila* photoreceptor cell development (Figure 2A) [125,130,132].

### 6.3. The K-RAS and SIAH Signaling Axis Is Highly Conserved in Cancer Biology

**Figure 3:**
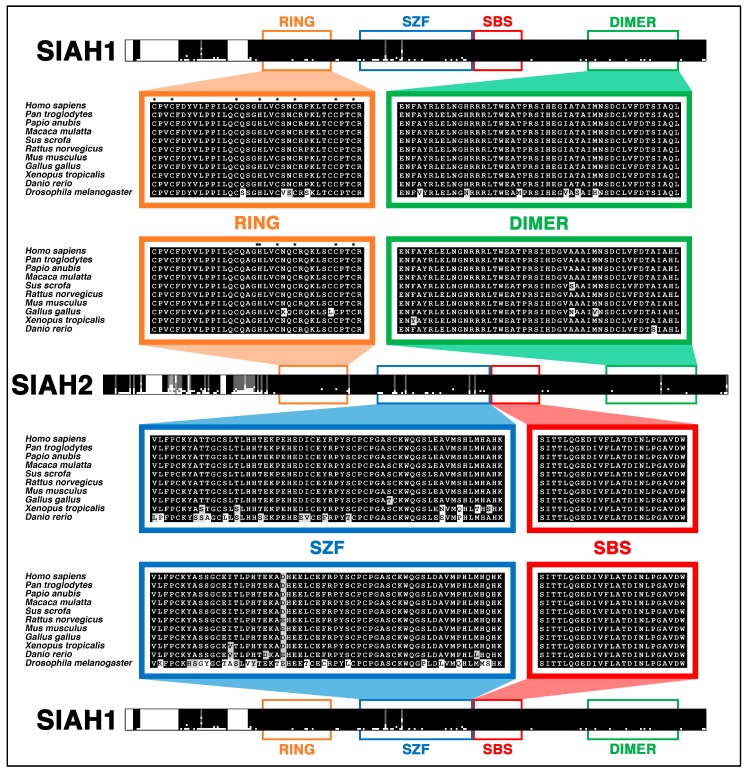
SIAH E3 ligases are highly evolutionarily conserved as shown by the extraordinary number of invariant amino acids in the sequences and four highly conserved functional domains (RING, SZF, SBS, and DIMER). The SIAH1 and SIAH2 sequences are compared and a large number of invariant amino acids are identified among the 11 most commonly used model species. These include ten vertebrate species–human, chimpanzee, baboon, macaque, pig, rat, mouse, chicken, frog, and zebra fish; and one invertebrate species–*Drosophila melanogaster*. MAFFT alignment of SIAH1 and SIAH2 amongst these model species reveals the extraordinarily high degree of evolutionary conservation in these SIAH1 and SIAH2 E3 ligases amongst vertebrate species. Furthermore, *Drosophila* SINA is also included in the vertebrate SIAH1 alignment, and the results show that even in invertebrate species, the SBS motif is identical, and the remaining functional domains of the SIAH family share an extraordinarily high degree of amino acid identity and/or similarity across the entire metazoan kingdom [73].

*Drosophila* SINA and two of its human homologs, SIAH1 and SIAH2, belong to an extraordinarily conserved family of RING domain E3 ligases that function as either homo- or hetero-dimers [73,145,146,147,148,149,150,151]. The SINA/SIAH family of E3 ligases have four distinct functional domains that are highly conserved: (1) the Really Interesting New Gene (RING) domain that functions as the catalytic active site for its E3 ligase activity; (2) the SIAH-type zinc finger (SZF) domain contains a dual zinc-finger motif; (3) the substrate-binding site (SBS) that recognizes and selects substrates; and (4) the dimerization (DIMER) domain that allows for homo- and heterodimerization between different SIAH proteins (Figure 3) [147,148,149,150,151,152,153,154,155]. The SZF, SBS, and DIMER domains form the substrate-binding domain (SBD), which is responsible for the recognition, interaction, targeting, and/or degradation of SIAH-interacting proteins (SIAH partners, regulators and substrates) [147,149,150,151,152,153,154,155]. SIAH E3 ligases are known to target and modify a multitude of diverse substrates, binding partners, and regulators. These interactions result in altered protein stability, context-dependent protein complex assembly, subcellular localization, and still other yet-to-be-identified cellular and molecular changes that influence normal development and tumor pathogenesis in vitro and in vivo [72,156,157]. As suggested by the *Drosophila* RAS studies, human SIAH-dependent proteolysis might also play a similar role as the most downstream signaling “gatekeeper” identified in the oncogenic K-RAS pathway in pancreatic cancer (Figure 2).

Mammalian SIAHs have been implicated in tumorigenesis by interacting with and modulating the stability of potent signaling molecules in oncogenesis; these include β-catenin, prolyl-hydroxylases/HIF-1α, TRAF2, NUMB, RINGO, Sprouty, and others [158,159,160,161,162,163,164,165,166]. However, the biological function, molecular regulation, substrate specificity, and targeting mechanism of SIAH E3 ligases in the context of constitutive K-RAS activation remains to be characterized in pancreatic cancer. The questions of how SIAH selectively interacts with its binding partners and degrades these substrates in response to oncogenic K-RAS activation, and how SIAH and newly identified SIAH substrates promote and facilitate oncogenic K-RAS-driven tumorigenesis and metastasis, remain to be elucidated in cancer biology.

Supported by extensive genetic studies of the RAS signal transduction pathway in *Drosophila,* its high degree of evolutionary conservation, and its strategic position as the most downstream signaling gatekeeper in the oncogenic K-RAS signaling pathway, SIAH is uniquely positioned to become the next generation anti-K-RAS drug target in pancreatic cancer (Figure 1 and Figure 2B). Our preclinical studies have demonstrated that SIAH-dependent proteolysis is a key tumor vulnerability and an “Achilles’ heel” for oncogenic K-RAS-driven high-grade and malignant tumors [72,134,135]. SIAH-insufficiency blocks oncogenic K-RAS signal transduction, and impedes pancreatic tumor formation in xenograft models [134]. Furthermore, SIAH expression in tumor cells reflects RAS pathway activation and tumor growth, whereas loss of SIAH expression in tumor cells reflects RAS pathway inactivation and tumor regression [134,135]. The clinical utility of SIAH as a therapy-responsive, prognostic and predictive biomarker has shown some promising potential in breast cancer in a retrospective pilot study [167,168]. Extensive clinical validation of the prognostic value of SIAH is still needed in high-risk and high-grade human cancer. Thus, developing an innovative anti-SIAH-based anti-K-RAS strategy will reveal novel SIAH pathway biomarker(s) as well as logical and potent SIAH-centered drug target(s) to eradicate and control pancreatic cancer in the future (Figure 2B).

### 6.4. SIAH Is Extraordinarily and Evolutionarily Conserved in Metazoa

By conducting phylogenetic analyses, we found that invertebrate SINA and vertebrate SIAH E3 ligases share an extraordinary degree of amino acid identity [73]. In this medically important gene family with its four highly conserved functional domains, SINA/SIAH has emerged as an extraordinarily conserved key signaling gatekeeper identified in the canonical EGFR/RAS/RAF/MEK/MAPK signal transduction cascade [73,169]. Phylogenetic analysis of this important group of RING domain SIAH E3 ligases has revealed many immutable amino acid residues and four critical functional domains that are highly conserved among all metazoan species (Figure 3). Thus, supported by strong evidence in developmental, evolutionary, and cancer biology, this phylogenetic analysis underscores the importance of evolution-based investigations in the conceptual design of logical and durable therapeutic strategies against the heretofore “undruggable” oncogenic K-RAS-driven pancreatic cancer [73].

Here we conducted phylogenetic analyses to align the amino acid sequences, identify the conserved structural motifs, and compare functional topology of invertebrate SINA, vertebrate SIAH1, and vertebrate SIAH2 proteins. We primarily focused on humans and ten model organisms commonly used in scientific investigations. Sequence homology analysis shows that the four distinct functional domains—the RING, SZF, SBS, and DIMER domains—exhibit an extraordinarily high degree of evolutionary conservation in vertebrate SIAH1, vertebrate SIAH2, and *Drosophila* SINA (Figure 3). All eight metal-coordinating cysteine (Cys) and histidine (His) residues are immutable amino acids in both the RING and SZF domains of these SIAH1/SIAH2/SINA proteins. Vertebrate SIAH2 has a unique N-terminal fragment that is longer than that of vertebrate SIAH1 (Figure 3). Despite this diverse N-terminal extension, the core consensus sequences of SIAH1 (amino acid #41-#282) and SIAH2 (amino acids #80-#324) share an extraordinarily high level of amino acid identity as shown in the protein alignment from 11 model species (Figure 3) [73].

This phylogenetic evidence provides an intellectual framework and a novel evolutionary perspective with which we can identify and dissect the invariant and divergent amino acid residues in the highly conserved functional domains of metazoan SINA/SIAH proteins that are required for their critical functions in transmitting the active RAS signal in human cancer. This is clinically relevant in the context of the SIAH1/2 gatekeeper function, as well as being a signaling bottleneck critical for oncogenic K-RAS activation, and a major tumor vulnerability in oncogenic K-RAS-driven human cancer [72,134,135]. The extraordinary degree of SINA/SIAH amino acid conservation is likely to provide invaluable insights into the design of next-generation SIAH-specific peptides and SIAH small molecule inhibitors as curative measures and durable therapeutics to control largely “undruggable” oncogenic K-RAS hyperactivation in PDAC. By following this line of SIAH-centered drug discovery and research investigation, we aim to design novel anti-SIAH strategies to target the major signaling hubs in the oncogenic K-RAS pathway, and develop innovative and curative measures to eradicate oncogenic K-RAS-driven malignant PDAC in the future.

### 6.5. SIAH Is a Tumor-Specific Biomarker in Human Pancreatic Cancer

**Figure 4:**
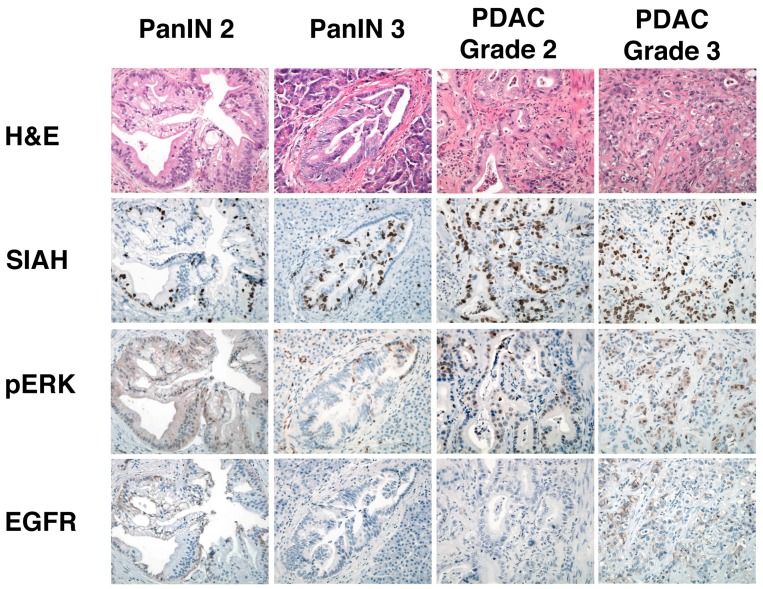
SIAH is a new tumor-specific biomarker in human pancreatic tumors. Representative images of the adjacent tumor areas that were serially sectioned and stained with H&E, SIAH, pERK and EGFR in pancreatic intraepithelial neoplasias (PanIN) and pancreatic ductal adenocarcinoma (PDAC) are shown. Note the specificity and clarity of SIAH staining in pancreatic tumor biospecimens. SIAH specifically decorates proliferating tumor cells in PanIN and PDAC. There is no SIAH staining in normal ductal epithelial cells, activated fibroblasts, infiltrating immune cells, and desmoplastic stromal tissues in the diseased pancreas. All the tumor images were captured under 400× magnification using a Leica DMR compound microscope.

Using anti-SIAH monoclonal antibodies (mAb), we examined the expression of SIAH in pancreatic intraepithelial neoplasias (PanIN) and pancreatic ductal adenocarcinoma (PDAC) [170]. Immunohistochemical (IHC) staining revealed that SIAH is expressed exclusively in proliferating tumor cells, while it is completely absent in the normal pancreas and nonmalignant TME cells (Figure 4). SIAH expression was predominantly nuclear and it increased with increased tumor grade and pathological stage (Figure 4). In comparison to several known biomarkers in the RAS signaling pathway, SIAH is a robust and tumor-specific biomarker that specifically decorates cycling pancreatic tumor cells. In contrast, there is a lack of consistency in tumor-specific staining of two main RAS pathway biomarkers: phospho-ERK sporadically decorated some tumor cells as well as some tumor-associated stromal cells, while EGFR decorated a small subset of PDAC tumor cells (Figure 4) [170].

### 6.6. A New Strategy of Anti-SIAH-Based Anti-K-RAS Therapy in Pancreatic Cancer

The central importance of aberrant K-RAS activation has been well established in human pancreatic cancer, where a vast majority of patients experience limited clinical options, rapid tumor relapse and metastasis, and largely dismal outcomes. Their need is urgent, and novel approaches to counteract activated K-RAS signals constitute important measures to reverse pancreatic tumorigenesis and metastasis. Instead of targeting an upstream signaling component such as EGFR/RAS/RAF/MEK, we propose to target the most downstream signaling module identified in the RAS pathway–the SIAH-dependent proteolytic machinery–a critical signaling gatekeeper required for K-RAS pathway signal transduction (Figure 1 and Figure 2). We have uncovered a logical, innovative, and effective strategy to shut down oncogenic K-RAS hyperactivation by attacking the most downstream signal transmission, SIAH, essential for proper K-RAS function. Further development of this evolution-based alternative anti-K-RAS approach for biomarker discovery and novel drug target validation is important for future clinical research. As an E3 ligase, SIAH should be druggable, as there are several successful precedents for developing small molecule inhibitors against the RING-domain and HECT-domain E3 ligases, including MDM2 (Nutlin-1, 2 and 3), APC, SCF-Skp2, and VHL [171,172,173,174,175,176,177,178,179,180,181,182]. Therefore, it is theoretically conceivable and practically plausible to screen for potent small molecule inhibitors against SIAH E3 ligases. If successful, the premise of developing specific SIAH inhibitors to control and eradicate human pancreatic cancer represents an exciting new area of cancer discovery.

In the past ten years, we have made crucial discoveries in uncovering the critical role of SIAH E3 ligase in the context of oncogenic K-RAS pathway activation that drives the genesis and progression of human pancreatic cancer. Our group in particular has previously made an important contribution in this area, as we were the first to present initial evidence demonstrating the importance of SIAH-dependent proteolysis in K-RAS-mediated tumorigenesis and metastasis in pancreatic cancer [134]. Our preclinical studies suggest that SIAH is a novel tumor-specific biomarker in human pancreatic cancer (Figure 4) [170]. As the most downstream and highly evolutionarily conserved signaling module, SIAH is ideally positioned to become the next-generation anti-K-RAS and anticancer drug target (Figure 1 and Figure 2). By inhibiting SIAH function, we have completely blocked tumorigenesis of MiaPaCa and Panc-1, two aggressive human PDAC cell lines, in athymic nude mice [134]. SIAH deficiency impairs K-RAS-mediated metastasis (extravasation) of human pancreatic cancer cells in xenograft models [183]. Our results have laid a solid foundation that clearly establishes an indispensable role of SIAH E3 ligases in oncogenic K-RAS signal transduction, neoplastic transformation, tumor growth and metastasis in human pancreatic cancer [134]. Supported by clear evidence from developmental, evolutionary, and cancer biology, the findings of these basic science studies have strong logic and high translational value to reshape the PDAC treatment landscape in the future. Conceptually, inhibiting SIAH function is an intuitive and logical way to inhibit oncogenic K-RAS activation and halt tumor growth and metastasis. It provides an alternative platform for the development of an oncogenic K-RAS signaling hub-centered and key PDAC tumor vulnerability-based intervention strategy for developing a logical “curative” therapeutic intervention in pancreatic cancer (Figure 2). Thus, SIAH is ideally positioned to emerge as a new, logical, phylomedicine-based therapeutic target against oncogenic K-RAS-driven malignant human cancers (Figure 1).

## 7. Future Perspectives

SIAH is ideally positioned to become a new therapeutic target for blocking constitutive K-RAS activation in pancreatic cancer [72,134]. We have successfully demonstrated that proper SIAH function is indispensable for oncogenic K-RAS signal transduction in human cancer [134,135]. A new anti-SIAH strategy will undoubtedly have an impact on oncogenic K-RAS signaling, pancreatic cancer biology, PDAC therapeutic intervention, and future drug development. Hence, advancing our understanding of SIAH in K-RAS signaling and, more importantly, exploring the potential of developing anti-SIAH inhibitors as anti-K-RAS and anti-PDAC agents may represent an exciting new area of cancer research.

Controlling and eradicating chemo-resistant, relapsed, and metastatic pancreatic cancer remains a major intellectual challenge and a pressing unmet need in science and medicine [79,82]. Supported by strong evidence in developmental and evolutionary biology, our group has demonstrated the efficacy of an alternative anticancer strategy by blocking the currently “undruggable” oncogenic K-RAS hyperactivation at its most downstream signaling gatekeeper, SIAH. We have demonstrated the irreversible effects of such a potent therapy in both in vitro and in vivo tumor models of oncogenic K-RAS-driven human pancreatic and lung cancer [134,135]. We and others have shown that anti-SIAH inhibitors were highly effective for diminishing oncogenic K-RAS/B-RAF activation and abolishing oncogenic K-RAS/B-RAF-driven tumorigenesis in xenograft models [134,135,156,184]. Phylogenetic analysis of this medicinally important family of SIAH RING-domain E3 ligases has shown us the evolutionary conservation and mutational constraints of invariant and divergent amino acid residues, as well as the 4 highly conserved functional domains in this metazoan SINA/SIAH family (Figure 3) [73]. This result provides a unique evolutionary, developmental, and cancer biology perspective supporting our central hypothesis that SIAH is a key signaling vulnerability in the oncogenic K-RAS pathway that is ripe for designing new anti-SIAH-based clinical interventions to shutdown oncogenic K-RAS-driven PDAC (Figure 1 and Figure 2). Similar to other E3 ligases with specific and potent inhibitors, SIAH E3 ligase should be druggable; as such, this anti-SIAH-based anti-K-RAS approach should have high impact and clear merit for rapid clinical translation.

Oncogenic K-RAS mutations are detected in about 95% of pancreatic cancers [84]. While a great deal of research has been done on K-RAS activation and RAS biology, there have only been a limited number of investigations to study SIAH biology, substrate selection, and targeting mechanisms. Thus, developing anti-SIAH-centered therapeutic strategies to achieve curative measures against oncogenic K-RAS-driven malignant cancer remains a relatively untapped and fertile area of research [72,134,135]. We found that SIAH expression is indicative of oncogenic K-RAS pathway activation and tumor progression, whereas the loss of SIAH expression is associated with K-RAS pathway inactivation and tumor regression [134,135,168,170]. Thus, focusing on this tumor-driving oncogenic K-RAS/SIAH pathway is a logical next step for not only clinically relevant biomarker discovery, but also for future development of new SIAH-centered anticancer therapies against oncogenic K-RAS driven pancreatic cancer. In the future, our K-RAS/SIAH pathway-centered biomarker discovery program may be used to stratify patients, identify chemo-refractory tumors, quantify tumor response and chemo-efficacy, forecast tumor relapse, and predict patient survival in resectable pancreatic cancer post-neoadjuvant chemotherapies in the clinic.

The field of oncogenic K-RAS biology and anti-K-RAS therapy is in desperate need of new breakthrough ideas, and curative measures to control and eradicate oncogenic K-RAS-driven pancreatic cancer [1,2,3,6,35]. SIAH-deficient oncogenic K-RAS-driven human cancer cells do not develop latent-phase tumor relapse in xenograft models, suggesting that such an anti-K-RAS blockade may be irreversible [134,135]. With our proposed new strategy of targeting SIAH E3 ligases–the most downstream signaling gatekeeper and critical signaling bottleneck in the oncogenic K-RAS signaling pathway, we may be in a position to overcome drug design obstacles such as compensatory pathway activation, cross talk, extensive signaling bifurcation, and dynamic feedback and feed-forward controls that are commonly observed in the K-RAS-driven tumors. Thus, our discovery of the role of SIAH in oncogenic K-RAS signal transduction in human pancreatic cancer represents a crucial conceptual innovation in the field of oncogenic K-RAS signaling and PDAC tumor biology (Figure 1 and Figure 2). As conventional standard therapies for high-grade and metastatic PDAC have proven largely ineffective, innovative approaches to block K-RAS activation by inhibiting SIAH enzymatic function may offer a viable alternative to provide tangible clinical benefits to more PDAC patients. Improving these irreversible and durable therapies may potentially lead to better tumor targeting and increased survival. In conclusion, we have proposed a new strategy to control “undruggable” K-RAS hyperactivation by targeting SIAH, a critical K-RAS signaling gatekeeper and a major tumor vulnerability in pancreatic cancer. By conducting chemical library screening in collaboration with our partners, we hope to find specific anti-SIAH molecules, as well as design SIAH peptides/small molecule inhibitors/proteolysis-deficient mutant SIAH (SIAH^PD^) as an alternative strategy to shut down oncogenic K-RAS hyperactivation, and abolish oncogenic K-RAS-driven tumorigenesis in preclinical and clinical models in the future. By simultaneously inhibiting active EGFR/RAS signaling at the upstream (EGFR), midstream (RAS/RAF/MEK/MAPK/AKT/mTOR) and downstream signaling modules (SIAH), these multipronged anti-K-RAS-centered combination therapies will work in tandem with chemo-, radiation, and other targeted therapies to increase the OS and PFS for pancreatic cancer patients who are in a desperate need of new curative therapies now and in the future.

## Abbreviations

DIMER	the dimerization domain
EGFR	epidermal growth factor receptor
ERK	Extracellular signal-Regulated Kinase
ETS	E-Twenty-Six
FTI	farnesyltransferase inhibitors
HER2	human epidermal growth factor receptor 2
HHMI	Howard Hughes Medical Institute
IHC	Immunohistochemical
MAPK	Mitogen-Activated Protein Kinase
MEK	Mitogen-Activated Protein Kinase kinase
NCI	National Cancer Institute
NIH	National Institutes of Health
OS	overall survival
PanCAN	Pancreatic Cancer Action Network
PanIN	pancreatic intraepithelial neoplasias
PDAC	pancreatic ductal adenocarcinoma
PDEδ	phosphodiesterase δ
PDX	patient-derived xenograft
PFS	progression free survival
RAF	RAF serine/threonine kinase
RAS	rat sarcoma viral oncogene
RING	Really Interesting New Gene
RTK	Receptor Tyrosine Kinase
SBD	substrate-binding domain
SBS	substrate-binding site
SEV	Sevenless receptor, the *Drosophila* homolog of mammalian EGFR membrane receptor
SIAH	Seven-In-Absentia Homolog
SINA	Seven-In-Absentia
SOC	standard of care
SOS	Son of Sevenless
SZF	SIAH-type zinc finger
TME	Tumor Microenvironment

## References

[1] Rahib L., Smith B.D., Aizenberg R., Rosenzweig A.B., Fleshman J.M., Matrisian L.M. (2014). Projecting cancer incidence and deaths to 2030: The unexpected burden of thyroid, liver, and pancreas cancers in the united states. Cancer Res..

[2] Garrido-Laguna I., Hidalgo M. (2015). Pancreatic cancer: From state-of-the-art treatments to promising novel therapies. Nat. Rev. Clin. Oncol..

[3] Kleeff J., Korc M., Apte M., La Vecchia C., Johnson C.D., Biankin A.V., Neale R.E., Tempero M., Tuveson D.A., Hruban R.H. (2016). Pancreatic cancer. Nat. Rev. Dis. Primers.

[4] Siegel R.L., Miller K.D., Jemal A. (2018). Cancer statistics, 2018. CA Cancer J. Clin..

[5] Bardeesy N., DePinho R.A. (2002). Pancreatic cancer biology and genetics. Nat. Rev. Cancer.

[6] Wolfgang C.L., Herman J.M., Laheru D.A., Klein A.P., Erdek M.A., Fishman E.K., Hruban R.H. (2013). Recent progress in pancreatic cancer. CA Cancer J. Clin..

[7] Sohn T.A., Yeo C.J., Cameron J.L., Koniaris L., Kaushal S., Abrams R.A., Sauter P.K., Coleman J., Hruban R.H., Lillemoe K.D. (2000). Resected adenocarcinoma of the pancreas-616 patients: Results, outcomes, and prognostic indicators. J. Gastrointest. Surg..

[8] Vincent A., Herman J., Schulick R., Hruban R.H., Goggins M. (2011). Pancreatic cancer. Lancet.

[9] Bockhorn M., Uzunoglu F.G., Adham M., Imrie C., Milicevic M., Sandberg A.A., Asbun H.J., Bassi C., Buchler M., Charnley R.M. (2014). Borderline resectable pancreatic cancer: A consensus statement by the international study group of pancreatic surgery (ISGPS). Surgery.

[10] Hidalgo M. (2010). Pancreatic cancer. N. Engl. J. Med..

[11] Strobel O., Hank T., Hinz U., Bergmann F., Schneider L., Springfeld C., Jager D., Schirmacher P., Hackert T., Buchler M.W. (2017). Pancreatic cancer surgery: The new r-status counts. Ann. Surg..

[12] Hruban R.H., Goggins M., Parsons J., Kern S.E. (2000). Progression model for pancreatic cancer. Clin. Cancer Res..

[13] Yeo T.P., Hruban R.H., Leach S.D., Wilentz R.E., Sohn T.A., Kern S.E., Iacobuzio-Donahue C.A., Maitra A., Goggins M., Canto M.I. (2002). Pancreatic cancer. Curr. Probl. Cancer.

[14] Katz M.H., Wang H., Fleming J.B., Sun C.C., Hwang R.F., Wolff R.A., Varadhachary G., Abbruzzese J.L., Crane C.H., Krishnan S. (2009). Long-term survival after multidisciplinary management of resected pancreatic adenocarcinoma. Ann. Surg. Oncol..

[15] Khorana A.A., Mangu P.B., Berlin J., Engebretson A., Hong T.S., Maitra A., Mohile S.G., Mumber M., Schulick R., Shapiro M. (2016). Potentially curable pancreatic cancer: American society of clinical oncology clinical practice guideline. J. Clin. Oncol..

[16] Rashid O.M., Pimiento J.M., Gamenthaler A.W., Nguyen P., Ha T.T., Hutchinson T., Springett G., Hoffe S., Shridhar R., Hodul P.J. (2016). Outcomes of a clinical pathway for borderline resectable pancreatic cancer. Ann. Surg. Oncol..

[17] Werner J., Combs S.E., Springfeld C., Hartwig W., Hackert T., Buchler M.W. (2013). Advanced-stage pancreatic cancer: Therapy options. Nat. Rev. Clin. Oncol..

[18] Sohal D.P., Mangu P.B., Khorana A.A., Shah M.A., Philip P.A., O’Reilly E.M., Uronis H.E., Ramanathan R.K., Crane C.H., Engebretson A. (2016). Metastatic pancreatic cancer: American society of clinical oncology clinical practice guideline. J. Clin. Oncol..

[19] Balaban E.P., Mangu P.B., Khorana A.A., Shah M.A., Mukherjee S., Crane C.H., Javle M.M., Eads J.R., Allen P., Ko A.H. (2016). Locally advanced, unresectable pancreatic cancer: American society of clinical oncology clinical practice guideline. J. Clin. Oncol..

[20] Conroy T., Desseigne F., Ychou M., Bouche O., Guimbaud R., Becouarn Y., Adenis A., Raoul J.L., Gourgou-Bourgade S., de la Fouchardiere C. (2011). Folfirinox versus gemcitabine for metastatic pancreatic cancer. N. Engl. J. Med..

[21] Valle J.W., Palmer D., Jackson R., Cox T., Neoptolemos J.P., Ghaneh P., Rawcliffe C.L., Bassi C., Stocken D.D., Cunningham D. (2014). Optimal duration and timing of adjuvant chemotherapy after definitive surgery for ductal adenocarcinoma of the pancreas: Ongoing lessons from the espac-3 study. J. Clin. Oncol..

[22] Von Hoff D.D., Ervin T., Arena F.P., Chiorean E.G., Infante J., Moore M., Seay T., Tjulandin S.A., Ma W.W., Saleh M.N. (2013). Increased survival in pancreatic cancer with nab-paclitaxel plus gemcitabine. N. Engl. J. Med..

[23] Oettle H., Post S., Neuhaus P., Gellert K., Langrehr J., Ridwelski K., Schramm H., Fahlke J., Zuelke C., Burkart C. (2007). Adjuvant chemotherapy with gemcitabine vs. observation in patients undergoing curative-intent resection of pancreatic cancer: A randomized controlled trial. JAMA.

[24] Tsai S., Evans D.B. (2016). Therapeutic advances in localized pancreatic cancer. JAMA Surg..

[25] Christians K.K., Heimler J.W., George B., Ritch P.S., Erickson B.A., Johnston F., Tolat P.P., Foley W.D., Evans D.B., Tsai S. (2016). Survival of patients with resectable pancreatic cancer who received neoadjuvant therapy. Surgery.

[26] Asare E.A., Evans D.B., Erickson B.A., Aburajab M., Tolat P., Tsai S. (2016). Neoadjuvant treatment sequencing adds value to the care of patients with operable pancreatic cancer. J. Surg. Oncol..

[27] Matrisian L.M., Berlin J.D. (2016). The past, present, and future of pancreatic cancer clinical trials. Am. Soc. Clin. Oncol. Educ. Book.

[28] Evans D.B., Erickson B.A., Ritch P. (2010). Borderline resectable pancreatic cancer: Definitions and the importance of multimodality therapy. Ann. Surg. Oncol..

[29] Boeck S., Haas M., Ormanns S., Kruger S., Siveke J.T., Heinemann V. (2014). Neoadjuvant chemotherapy in pancreatic cancer: Innovative, but still difficult. Br. J. Cancer.

[30] Evans D.B., Ritch P.S., Erickson B.A. (2015). Neoadjuvant therapy for localized pancreatic cancer: Support is growing?. Ann. Surg..

[31] Canto M.I., Hruban R.H. (2015). Diagnosis: A step closer to screening for curable pancreatic cancer?. Nat. Rev. Gastroenterol. Hepatol..

[32] Lennon A.M., Wolfgang C.L., Canto M.I., Klein A.P., Herman J.M., Goggins M., Fishman E.K., Kamel I., Weiss M.J., Diaz L.A. (2014). The early detection of pancreatic cancer: What will it take to diagnose and treat curable pancreatic neoplasia?. Cancer Res..

[33] Makohon-Moore A., Iacobuzio-Donahue C.A. (2016). Pancreatic cancer biology and genetics from an evolutionary perspective. Nat. Rev. Cancer.

[34] Rahib L., Fleshman J.M., Matrisian L.M., Berlin J.D. (2016). Evaluation of pancreatic cancer clinical trials and benchmarks for clinically meaningful future trials: A systematic review. JAMA Oncol..

[35] Maitra A., Hruban R.H. (2008). Pancreatic cancer. Annu. Rev. Pathol..

[36] Pishvaian M.J., Joseph Bender R., Matrisian L.M., Rahib L., Hendifar A., Hoos W.A., Mikhail S., Chung V., Picozzi V., Heartwell C. (2017). A pilot study evaluating concordance between blood-based and patient-matched tumor molecular testing within pancreatic cancer patients participating in the know your tumor (KYT) initiative. Oncotarget.

[37] Pylayeva-Gupta Y., Grabocka E., Bar-Sagi D. (2011). Ras oncogenes: Weaving a tumorigenic web. Nat. Rev. Cancer.

[38] Waddell N., Pajic M., Patch A.M., Chang D.K., Kassahn K.S., Bailey P., Johns A.L., Miller D., Nones K., Quek K. (2015). Whole genomes redefine the mutational landscape of pancreatic cancer. Nature.

[39] Witkiewicz A.K., McMillan E.A., Balaji U., Baek G., Lin W.C., Mansour J., Mollaee M., Wagner K.U., Koduru P., Yopp A. (2015). Whole-exome sequencing of pancreatic cancer defines genetic diversity and therapeutic targets. Nat. Commun..

[40] Makohon-Moore A.P., Zhang M., Reiter J.G., Bozic I., Allen B., Kundu D., Chatterjee K., Wong F., Jiao Y., Kohutek Z.A. (2017). Limited heterogeneity of known driver gene mutations among the metastases of individual patients with pancreatic cancer. Nat. Genet..

[41] Yachida S., Jones S., Bozic I., Antal T., Leary R., Fu B., Kamiyama M., Hruban R.H., Eshleman J.R., Nowak M.A. (2010). Distant metastasis occurs late during the genetic evolution of pancreatic cancer. Nature.

[42] Campbell P.J., Yachida S., Mudie L.J., Stephens P.J., Pleasance E.D., Stebbings L.A., Morsberger L.A., Latimer C., McLaren S., Lin M.L. (2010). The patterns and dynamics of genomic instability in metastatic pancreatic cancer. Nature.

[43] Viale A., Pettazzoni P., Lyssiotis C.A., Ying H., Sanchez N., Marchesini M., Carugo A., Green T., Seth S., Giuliani V. (2014). Oncogene ablation-resistant pancreatic cancer cells depend on mitochondrial function. Nature.

[44] Yang A., Herter-Sprie G., Zhang H., Lin E.Y., Biancur D., Wang X., Deng J., Hai J., Yang S., Wong K.K. (2018). Autophagy sustains pancreatic cancer growth through both cell autonomous and non-autonomous mechanisms. Cancer Discov..

[45] Kai F., Laklai H., Weaver V.M. (2016). Force matters: Biomechanical regulation of cell invasion and migration in disease. Trends Cell Biol..

[46] Laklai H., Miroshnikova Y.A., Pickup M.W., Collisson E.A., Kim G.E., Barrett A.S., Hill R.C., Lakins J.N., Schlaepfer D.D., Mouw J.K. (2016). Genotype tunes pancreatic ductal adenocarcinoma tissue tension to induce matricellular fibrosis and tumor progression. Nat. Med..

[47] Vakoc C.R., Tuveson D.A. (2017). Untangling the genetics from the epigenetics in pancreatic cancer metastasis. Nat. Genet..

[48] Brahmer J.R., Tykodi S.S., Chow L.Q., Hwu W.J., Topalian S.L., Hwu P., Drake C.G., Camacho L.H., Kauh J., Odunsi K. (2012). Safety and activity of anti-PD-L1 antibody in patients with advanced cancer. N. Engl. J. Med..

[49] Royal R.E., Levy C., Turner K., Mathur A., Hughes M., Kammula U.S., Sherry R.M., Topalian S.L., Yang J.C., Lowy I. (2010). Phase 2 trial of single agent ipilimumab (anti-CTLA-4) for locally advanced or metastatic pancreatic adenocarcinoma. J. Immunother..

[50] Kunk P.R., Bauer T.W., Slingluff C.L., Rahma O.E. (2016). From bench to bedside a comprehensive review of pancreatic cancer immunotherapy. J. Immunother. Cancer.

[51] Sharma P., Allison J.P. (2015). The future of immune checkpoint therapy. Science.

[52] Laheru D., Jaffee E.M. (2005). Immunotherapy for pancreatic cancer—Science driving clinical progress. Nat. Rev. Cancer.

[53] Olive K.P., Jacobetz M.A., Davidson C.J., Gopinathan A., McIntyre D., Honess D., Madhu B., Goldgraben M.A., Caldwell M.E., Allard D. (2009). Inhibition of hedgehog signaling enhances delivery of chemotherapy in a mouse model of pancreatic cancer. Science.

[54] Rhim A.D., Oberstein P.E., Thomas D.H., Mirek E.T., Palermo C.F., Sastra S.A., Dekleva E.N., Saunders T., Becerra C.P., Tattersall I.W. (2014). Stromal elements act to restrain, rather than support, pancreatic ductal adenocarcinoma. Cancer Cell.

[55] Twyman-Saint Victor C., Rech A.J., Maity A., Rengan R., Pauken K.E., Stelekati E., Benci J.L., Xu B., Dada H., Odorizzi P.M. (2015). Radiation and dual checkpoint blockade activate non-redundant immune mechanisms in cancer. Nature.

[56] Winograd R., Byrne K.T., Evans R.A., Odorizzi P.M., Meyer A.R., Bajor D.L., Clendenin C., Stanger B.Z., Furth E.E., Wherry E.J. (2015). Induction of T-cell immunity overcomes complete resistance to PD-1 and CTLA-4 blockade and improves survival in pancreatic carcinoma. Cancer Immunol. Res..

[57] Byrne K.T., Vonderheide R.H. (2016). CD40 stimulation obviates innate sensors and drives t cell immunity in cancer. Cell Rep..

[58] Vonderheide R.H. (2015). CD47 blockade as another immune checkpoint therapy for cancer. Nat. Med..

[59] Vonderheide R.H., Bajor D.L., Winograd R., Evans R.A., Bayne L.J., Beatty G.L. (2013). CD40 immunotherapy for pancreatic cancer. Cancer Immunol. Immunother..

[60] Vonderheide R.H., Bayne L.J. (2013). Inflammatory networks and immune surveillance of pancreatic carcinoma. Curr. Opin. Immunol..

[61] Beatty G.L., Torigian D.A., Chiorean E.G., Saboury B., Brothers A., Alavi A., Troxel A.B., Sun W., Teitelbaum U.R., Vonderheide R.H. (2013). A phase i study of an agonist CD40 monoclonal antibody (cp-870,893) in combination with gemcitabine in patients with advanced pancreatic ductal adenocarcinoma. Clin. Cancer Res..

[62] Beatty G.L., Chiorean E.G., Fishman M.P., Saboury B., Teitelbaum U.R., Sun W., Huhn R.D., Song W., Li D., Sharp L.L. (2011). CD40 agonists alter tumor stroma and show efficacy against pancreatic carcinoma in mice and humans. Science.

[63] Chakrabarti G., Silvers M.A., Ilcheva M., Liu Y., Moore Z.R., Luo X., Gao J., Anderson G., Liu L., Sarode V. (2015). Tumor-selective use of DNA base excision repair inhibition in pancreatic cancer using the nqo1 bioactivatable drug, beta-lapachone. Sci. Rep..

[64] Beg M.S., Huang X., Silvers M.A., Gerber D.E., Bolluyt J., Sarode V., Fattah F., Deberardinis R.J., Merritt M.E., Xie X.J. (2017). Using a novel NQO1 bioactivatable drug, β-Lapachone (arq761), to enhance chemotherapeutic effects by metabolic modulation in pancreatic cancer. J. Surg. Oncol..

[65] Tempero M. (2016). Pancreatic adenocarcinoma: The emperor of all cancer maladies. J. Oncol. Pract..

[66] Barbacid M. (1987). Ras genes. Annu. Rev. Biochem..

[67] Jones S., Zhang X., Parsons D.W., Lin J.C., Leary R.J., Angenendt P., Mankoo P., Carter H., Kamiyama H., Jimeno A. (2008). Core signaling pathways in human pancreatic cancers revealed by global genomic analyses. Science.

[68] Vogelstein B., Papadopoulos N., Velculescu V.E., Zhou S., Diaz L.A., Kinzler K.W. (2013). Cancer genome landscapes. Science.

[69] Cox A.D., Fesik S.W., Kimmelman A.C., Luo J., Der C.J. (2014). Drugging the undruggable ras: Mission possible?. Nat. Rev. Drug Discov..

[70] Downward J. (2003). Targeting ras signalling pathways in cancer therapy. Nat. Rev. Cancer.

[71] Hanahan D., Weinberg R.A. (2011). Hallmarks of cancer: The next generation. Cell.

[72] Van Sciver R.E., Njogu M.M., Isbell A.J., Odanga J.J., Bian M., Svyatova E., van Reesema L.L.S., Zheleva V., Eisner J.L., Bruflat J.K., Azmi A.S. (2016). Blocking siah proteolysis, an important K-RAS vulnerability, to control and eradicate K-RAS-driven metastatic cancer. Conquering RAS: From Biology to Cancer Therapy.

[73] Pepper I.J., Van Sciver R.E., Tang A.H. (2017). Phylogenetic analysis of the SINA/SIAH ubiquitin E3 ligase family in metazoa. BMC Evol. Biol..

[74] Lowy D.R., Willumsen B.M. (1993). Function and regulation of ras. Annu. Rev. Biochem..

[75] Hynes N.E., Lane H.A. (2005). ERBB receptors and cancer: The complexity of targeted inhibitors. Nat. Rev. Cancer.

[76] Gschwind A., Fischer O.M., Ullrich A. (2004). The discovery of receptor tyrosine kinases: Targets for cancer therapy. Nat. Rev. Cancer.

[77] Schubbert S., Shannon K., Bollag G. (2007). Hyperactive ras in developmental disorders and cancer. Nat. Rev. Cancer.

[78] Stephen A.G., Esposito D., Bagni R.K., McCormick F. (2014). Dragging RAS back in the ring. Cancer Cell.

[79] McCormick F. (2015). Kras as a therapeutic target. Clin. Cancer Res..

[80] Haigis K.M. (2017). Kras alleles: The devil is in the detail. Trends Cancer.

[81] Bos J.L. (1989). Ras oncogenes in human cancer: A review. Cancer Res..

[82] Malumbres M., Barbacid M. (2003). Ras oncogenes: The first 30 years. Nat. Rev. Cancer.

[83] Simanshu D.K., Nissley D.V., McCormick F. (2017). Ras proteins and their regulators in human disease. Cell.

[84] Bailey P., Chang D.K., Nones K., Johns A.L., Patch A.M., Gingras M.C., Miller D.K., Christ A.N., Bruxner T.J., Quinn M.C. (2016). Genomic analyses identify molecular subtypes of pancreatic cancer. Nature.

[85] Hanahan D., Weinberg R.A. (2000). The hallmarks of cancer. Cell.

[86] Sebolt-Leopold J.S., Herrera R. (2004). Targeting the mitogen-activated protein kinase cascade to treat cancer. Nat. Rev. Cancer.

[87] Chambers A.F., Groom A.C., MacDonald I.C. (2002). Dissemination and growth of cancer cells in metastatic sites. Nat. Rev. Cancer.

[88] Pagliarini R.A., Xu T. (2003). A genetic screen in drosophila for metastatic behavior. Science.

[89] Giehl K. (2005). Oncogenic ras in tumour progression and metastasis. Biol. Chem..

[90] Koop S., Schmidt E.E., MacDonald I.C., Morris V.L., Khokha R., Grattan M., Leone J., Chambers A.F., Groom A.C. (1996). Independence of metastatic ability and extravasation: Metastatic RAS-transformed and control fibroblasts extravasate equally well. Proc. Natl. Acad. Sci. USA.

[91] Varghese H.J., Davidson M.T., MacDonald I.C., Wilson S.M., Nadkarni K.V., Groom A.C., Chambers A.F. (2002). Activated RAS regulates the proliferation/apoptosis balance and early survival of developing micrometastases. Cancer Res..

[92] Gibbs J.B., Oliff A., Kohl N.E. (1994). Farnesyltransferase inhibitors: RAS research yields a potential cancer therapeutic. Cell.

[93] Kohl N.E., Wilson F.R., Mosser S.D., Giuliani E., deSolms S.J., Conner M.W., Anthony N.J., Holtz W.J., Gomez R.P., Lee T.J. (1994). Protein farnesyltransferase inhibitors block the growth of RAS-dependent tumors in nude mice. Proc. Natl. Acad. Sci. USA.

[94] Gibbs J.B., Graham S.L., Hartman G.D., Koblan K.S., Kohl N.E., Omer C.A., Oliff A. (1997). Farnesyltransferase inhibitors versus ras inhibitors. Curr. Opin. Chem. Biol..

[95] Caponigro F., Casale M., Bryce J. (2003). Farnesyl transferase inhibitors in clinical development. Expert Opin. Investig. Drugs.

[96] Baines A.T., Xu D., Der C.J. (2011). Inhibition of RAS for cancer treatment: The search continues. Future Med. Chem..

[97] Appels N.M., Beijnen J.H., Schellens J.H. (2005). Development of farnesyl transferase inhibitors: A review. Oncologist.

[98] Papke B., Murarka S., Vogel H.A., Martin-Gago P., Kovacevic M., Truxius D.C., Fansa E.K., Ismail S., Zimmermann G., Heinelt K. (2016). Identification of pyrazolopyridazinones as pdedelta inhibitors. Nat. Commun..

[99] Chen L., Zhuang C., Lu J., Jiang Y., Sheng C. (2018). Discovery of novel kras-pdedelta inhibitors by fragment-based drug design. J. Med. Chem..

[100] Zimmermann G., Papke B., Ismail S., Vartak N., Chandra A., Hoffmann M., Hahn S.A., Triola G., Wittinghofer A., Bastiaens P.I. (2013). Small molecule inhibition of the KRAS-PDEδ interaction impairs oncogenic KRAS signalling. Nature.

[101] Hobbs G.A., Wittinghofer A., Der C.J. (2016). Selective targeting of the KRAS g12c mutant: Kicking KRAS when it’s down. Cancer Cell.

[102] Ostrem J.M., Peters U., Sos M.L., Wells J.A., Shokat K.M. (2013). K-RAS(g12c) inhibitors allosterically control GTP affinity and effector interactions. Nature.

[103] Hunter J.C., Gurbani D., Ficarro S.B., Carrasco M.A., Lim S.M., Choi H.G., Xie T., Marto J.A., Chen Z., Gray N.S. (2014). In situ selectivity profiling and crystal structure of SML-8-73-1, an active site inhibitor of oncogenic K-RAS g12c. Proc. Natl. Acad. Sci. USA.

[104] Lim S.M., Westover K.D., Ficarro S.B., Harrison R.A., Choi H.G., Pacold M.E., Carrasco M., Hunter J., Kim N.D., Xie T. (2014). Therapeutic targeting of oncogenic K-RAS by a covalent catalytic site inhibitor. Angew. Chem. Int. Ed. Engl..

[105] Lito P., Solomon M., Li L.S., Hansen R., Rosen N. (2016). Allele-specific inhibitors inactivate mutant KRAS g12c by a trapping mechanism. Science.

[106] Patricelli M.P., Janes M.R., Li L.S., Hansen R., Peters U., Kessler L.V., Chen Y., Kucharski J.M., Feng J., Ely T. (2016). Selective inhibition of oncogenic KRAS output with small molecules targeting the inactive state. Cancer Discov..

[107] Westover K.D., Janne P.A., Gray N.S. (2016). Progress on covalent inhibition of KRAS(g12c). Cancer Discov..

[108] Muller M.P., Jeganathan S., Heidrich A., Campos J., Goody R.S. (2017). Nucleotide based covalent inhibitors of kras can only be efficient in vivo if they bind reversibly with GTP-like affinity. Sci. Rep..

[109] Janes M.R., Zhang J., Li L.S., Hansen R., Peters U., Guo X., Chen Y., Babbar A., Firdaus S.J., Darjania L. (2018). Targeting KRAS mutant cancers with a covalent g12c-specific inhibitor. Cell.

[110] Hobbs G.A., Der C.J., Rossman K.L. (2016). Ras isoforms and mutations in cancer at a glance. J. Cell Sci..

[111] Edkins S., O’Meara S., Parker A., Stevens C., Reis M., Jones S., Greenman C., Davies H., Dalgliesh G., Forbes S. (2006). Recurrent KRAS codon 146 mutations in human colorectal cancer. Cancer Biol. Ther..

[112] Imamura Y., Lochhead P., Yamauchi M., Kuchiba A., Qian Z.R., Liao X., Nishihara R., Jung S., Wu K., Nosho K. (2014). Analyses of clinicopathological, molecular, and prognostic associations of KRAS codon 61 and codon 146 mutations in colorectal cancer: Cohort study and literature review. Mol. Cancer.

[113] McCormick F. (2016). K-ras protein as a drug target. J. Mol. Med..

[114] Yachida S., White C.M., Naito Y., Zhong Y., Brosnan J.A., Macgregor-Das A.M., Morgan R.A., Saunders T., Laheru D.A., Herman J.M. (2012). Clinical significance of the genetic landscape of pancreatic cancer and implications for identification of potential long-term survivors. Clin. Cancer Res..

[115] Nowell P.C. (1976). The clonal evolution of tumor cell populations. Science.

[116] Niederhuber J.E. (2007). Developmental biology, self-renewal, and cancer. Lancet Oncol..

[117] Greaves M., Maley C.C. (2012). Clonal evolution in cancer. Nature.

[118] Gluckman P.D., Beedle A., Hanson M.A. (2009). Principles of Evolutionary Medicine.

[119] Edwards P.A. (1999). The impact of developmental biology on cancer research: An overview. Cancer Metastasis Rev..

[120] Xie K., Abbruzzese J.L. (2003). Developmental biology informs cancer: The emerging role of the hedgehog signaling pathway in upper gastrointestinal cancers. Cancer Cell.

[121] Frank S.A., Nowak M.A. (2003). Cell biology: Developmental predisposition to cancer. Nature.

[122] Pepper J.W., Scott Findlay C., Kassen R., Spencer S.L., Maley C.C. (2009). Cancer research meets evolutionary biology. Evol. Appl..

[123] Greenwald I., Rubin G.M. (1992). Making a difference: The role of cell-cell interactions in establishing separate identities for equivalent cells. Cell.

[124] Rubin G.M., Chang H.C., Karim F., Laverty T., Michaud N.R., Morrison D.K., Rebay I., Tang A., Therrien M., Wassarman D.A. (1997). Signal transduction downstream from RAS in drosophila. Cold Spring Harbor Symposia on Quantitative Biology.

[125] Zipursky S.L., Rubin G.M. (1994). Determination of neuronal cell fate: Lessons from the R7 neuron of drosophila. Annu. Rev. Neurosci..

[126] Mackay T.F. (2014). Epistasis and quantitative traits: Using model organisms to study gene-gene interactions. Nat. Rev. Genet..

[127] Sackton T.B., Hartl D.L. (2016). Genotypic context and epistasis in individuals and populations. Cell.

[128] Carthew R.W., Rubin G.M. (1990). Seven in absentia, a gene required for specification of R7 cell fate in the drosophila eye. Cell.

[129] Dickson B., Hafen E. (1994). Genetics of signal transduction in invertebrates. Curr. Opin. Genet. Dev..

[130] Fortini M.E., Simon M.A., Rubin G.M. (1992). Signaling by the sevenless protein tyrosine kinase is mimicked by RAS1 activation. Nature.

[131] Simon M.A., Bowtell D.D., Dodson G.S., Laverty T.R., Rubin G.M. (1991). RAS1 and a putative guanine nucleotide exchange factor perform crucial steps in signaling by the sevenless protein tyrosine kinase. Cell.

[132] Wassarman D.A., Therrien M., Rubin G.M. (1995). The RAS signaling pathway in drosophila. Curr. Opin. Genet. Dev..

[133] Raabe T. (2000). The sevenless signaling pathway: Variations of a common theme. Biochim. Biophys. Acta.

[134] Schmidt R.L., Park C.H., Ahmed A.U., Gundelach J.H., Reed N.R., Cheng S., Knudsen B.E., Tang A.H. (2007). Inhibition of RAS-mediated transformation and tumorigenesis by targeting the downstream E3 ubiquitin ligase seven in absentia homologue. Cancer Res..

[135] Ahmed A.U., Schmidt R.L., Park C.H., Reed N.R., Hesse S.E., Thomas C.F., Molina J.R., Deschamps C., Yang P., Aubry M.C. (2008). Effect of disrupting seven-in-absentia homolog 2 function on lung cancer cell growth. J. Natl. Cancer Inst..

[136] Widmann C., Gibson S., Jarpe M.B., Johnson G.L. (1999). Mitogen-activated protein kinase: Conservation of a three-kinase module from yeast to human. Physiol. Rev..

[137] Cox A.D., Der C.J. (2010). Ras history: The saga continues. Small GTPases.

[138] Rojas A.M., Fuentes G., Rausell A., Valencia A. (2012). The RAS protein superfamily: Evolutionary tree and role of conserved amino acids. J. Cell Biol..

[139] Slack C. (2017). Ras signaling in aging and metabolic regulation. Nutr. Healthy Aging.

[140] Xu C., Liu R., Zhang Q., Chen X., Qian Y., Fang W. (2016). The diversification of evolutionarily conserved mapk cascades correlates with the evolution of fungal species and development of lifestyles. Genome Biol. Evol..

[141] Simon M.A., Bowtell D.D., Rubin G.M. (1989). Structure and activity of the sevenless protein: A protein tyrosine kinase receptor required for photoreceptor development in drosophila. Proc. Natl. Acad. Sci. USA.

[142] Dickson B., Sprenger F., Morrison D., Hafen E. (1992). Raf functions downstream of RAS1 in the sevenless signal transduction pathway. Nature.

[143] Brunner D., Oellers N., Szabad J., Biggs W.H., Zipursky S.L., Hafen E. (1994). A gain-of-function mutation in drosophila map kinase activates multiple receptor tyrosine kinase signaling pathways. Cell.

[144] Biggs W.H., Zavitz K.H., Dickson B., van der Straten A., Brunner D., Hafen E., Zipursky S.L. (1994). The drosophila rolled locus encodes a map kinase required in the sevenless signal transduction pathway. EMBO J..

[145] Tang A.H., Neufeld T.P., Kwan E., Rubin G.M. (1997). PHYL acts to down-regulate TTK88, a transcriptional repressor of neuronal cell fates, by a SINA-dependent mechanism. Cell.

[146] Hu G., Chung Y.L., Glover T., Valentine V., Look A.T., Fearon E.R. (1997). Characterization of human homologs of the drosophila seven in absentia (SINA) gene. Genomics.

[147] Zhang Q., Wang Z., Hou F., Harding R., Huang X., Dong A., Walker J.R., Tong Y. (2017). The substrate binding domains of human SIAH E3 ubiquitin ligases are now crystal clear. Biochim. Biophys. Acta.

[148] Mei Y., Xie C., Xie W., Wu Z., Wu M. (2007). SIAH-1s, a novel splice variant of SIAH-1 (seven in absentia homolog), counteracts SIAH-1-mediated downregulation of β-catenin. Oncogene.

[149] Polekhina G., House C.M., Traficante N., Mackay J.P., Relaix F., Sassoon D.A., Parker M.W., Bowtell D.D. (2002). SIAH ubiquitin ligase is structurally related to TRAF and modulates TNF-α signaling. Nat. Struct. Biol..

[150] Matsuzawa S., Li C., Ni C.Z., Takayama S., Reed J.C., Ely K.R. (2003). Structural analysis of SIAH1 and its interactions with siah-interacting protein (SIP). J. Biol. Chem..

[151] House C.M., Hancock N.C., Moller A., Cromer B.A., Fedorov V., Bowtell D.D., Parker M.W., Polekhina G. (2006). Elucidation of the substrate binding site of siah ubiquitin ligase. Structure.

[152] Hu G., Fearon E.R. (1999). Siah-1 n-terminal ring domain is required for proteolysis function, and C-terminal sequences regulate oligomerization and binding to target proteins. Mol. Cell. Biol..

[153] Reed J.C., Ely K.R. (2002). Degrading liaisons: SIAH structure revealed. Nat. Struct. Biol..

[154] Santelli E., Leone M., Li C., Fukushima T., Preece N.E., Olson A.J., Ely K.R., Reed J.C., Pellecchia M., Liddington R.C. (2005). Structural analysis of SIAH1-SIAH-interacting protein interactions and insights into the assembly of an E3 ligase multiprotein complex. J. Biol. Chem..

[155] Depaux A., Regnier-Ricard F., Germani A., Varin-Blank N. (2006). Dimerization of hsiah proteins regulates their stability. Biochem. Biophys. Res. Commun..

[156] Moller A., House C.M., Wong C.S., Scanlon D.B., Liu M.C., Ronai Z., Bowtell D.D. (2009). Inhibition of SIAH ubiquitin ligase function. Oncogene.

[157] Qi J., Kim H., Scortegagna M., Ronai Z.A. (2013). Regulators and effectors of siah ubiquitin ligases. Cell Biochem. Biophys..

[158] Nadeau R.J., Toher J.L., Yang X., Kovalenko D., Friesel R. (2007). Regulation of sprouty2 stability by mammalian seven-in-absentia homolog 2. J. Cell. Biochem..

[159] Sutterluty H., Mayer C.E., Setinek U., Attems J., Ovtcharov S., Mikula M., Mikulits W., Micksche M., Berger W. (2007). Down-regulation of sprouty2 in non-small cell lung cancer contributes to tumor malignancy via extracellular signal-regulated kinase pathway-dependent and -independent mechanisms. Mol. Cancer Res..

[160] Kim H.J., Taylor L.J., Bar-Sagi D. (2007). Spatial regulation of egfr signaling by sprouty2. Curr. Biol..

[161] Shaw A.T., Meissner A., Dowdle J.A., Crowley D., Magendantz M., Ouyang C., Parisi T., Rajagopal J., Blank L.J., Bronson R.T. (2007). Sprouty-2 regulates oncogenic K-RAS in lung development and tumorigenesis. Genes Dev..

[162] Gutierrez G.J., Vogtlin A., Castro A., Ferby I., Salvagiotto G., Ronai Z., Lorca T., Nebreda A.R. (2006). Meiotic regulation of the cdk activator ringo/speedy by ubiquitin-proteasome-mediated processing and degradation. Nat. Cell Biol..

[163] Habelhah H., Frew I.J., Laine A., Janes P.W., Relaix F., Sassoon D., Bowtell D.D., Ronai Z. (2002). Stress-induced decrease in TRAF2 stability is mediated by SIAH2. EMBO J..

[164] Nakayama K., Frew I.J., Hagensen M., Skals M., Habelhah H., Bhoumik A., Kadoya T., Erdjument-Bromage H., Tempst P., Frappell P.B. (2004). SIAH2 regulates stability of prolyl-hydroxylases, controls HIF1α abundance, and modulates physiological responses to hypoxia. Cell.

[165] Matsuzawa S.I., Reed J.C. (2001). SIAH-1, SIP, and EBI collaborate in a novel pathway for β-catenin degradation linked to p53 responses. Mol. Cell.

[166] Susini L., Passer B.J., Amzallag-Elbaz N., Juven-Gershon T., Prieur S., Privat N., Tuynder M., Gendron M.C., Israel A., Amson R. (2001). SIAH-1 binds and regulates the function of numb. Proc. Natl. Acad. Sci. USA.

[167] Behling K.C., Tang A., Freydin B., Chervoneva I., Kadakia S., Schwartz G.F., Rui H., Witkiewicz A.K. (2011). Increased SIAH expression predicts ductal carcinoma in situ (DCIS) progression to invasive carcinoma. Breast Cancer Res. Treat..

[168] Van Reesema L.L., Zheleva V., Winston J.S., Jansen R.J., O’Connor C.F., Isbell A.J., Bian M., Qin R., Bassett P.T., Hinson V.J. (2016). SIAH and EGFR, two ras pathway biomarkers, are highly prognostic in locally advanced and metastatic breast cancer. EBioMedicine.

[169] Hu G., Zhang S., Vidal M., Baer J.L., Xu T., Fearon E.R. (1997). Mammalian homologs of seven in absentia regulate DCC via the ubiquitin-proteasome pathway. Genes Dev..

[170] Qin R., Smyrk T.C., Reed N.R., Schmidt R.L., Schnelldorfer T., Chari S.T., Petersen G.M., Tang A.H. (2015). Combining clinicopathological predictors and molecular biomarkers in the oncogenic K-RAS/KI67/HIF-1α pathway to predict survival in resectable pancreatic cancer. Br. J. Cancer.

[171] Huang J., Sheung J., Dong G., Coquilla C., Daniel-Issakani S., Payan D.G. (2005). High-throughput screening for inhibitors of the E3 ubiquitin ligase APC. Methods Enzymol..

[172] Herman A.G., Hayano M., Poyurovsky M.V., Shimada K., Skouta R., Prives C., Stockwell B.R. (2011). Discovery of MDM2-MDMX E3 ligase inhibitors using a cell-based ubiquitination assay. Cancer Discov..

[173] Yagishita N., Aratani S., Leach C., Amano T., Yamano Y., Nakatani K., Nishioka K., Nakajima T. (2012). Ring-finger type E3 ubiquitin ligase inhibitors as novel candidates for the treatment of rheumatoid arthritis. Int. J. Mol. Med..

[174] Rossi M., Rotblat B., Ansell K., Amelio I., Caraglia M., Misso G., Bernassola F., Cavasotto C.N., Knight R.A., Ciechanover A. (2014). High throughput screening for inhibitors of the HECT ubiquitin E3 ligase ITCH identifies antidepressant drugs as regulators of autophagy. Cell Death Dis..

[175] Landre V., Rotblat B., Melino S., Bernassola F., Melino G. (2014). Screening for E3-ubiquitin ligase inhibitors: Challenges and opportunities. Oncotarget.

[176] Brummelkamp T.R., Fabius A.W., Mullenders J., Madiredjo M., Velds A., Kerkhoven R.M., Bernards R., Beijersbergen R.L. (2006). An shRNA barcode screen provides insight into cancer cell vulnerability to MDM2 inhibitors. Nat. Chem. Biol..

[177] Vassilev L.T., Vu B.T., Graves B., Carvajal D., Podlaski F., Filipovic Z., Kong N., Kammlott U., Lukacs C., Klein C. (2004). In Vivo activation of the p53 pathway by small-molecule antagonists of MDM2. Science.

[178] Vassilev L.T. (2007). MDM2 inhibitors for cancer therapy. Trends Mol. Med..

[179] Shangary S., Wang S. (2009). Small-molecule inhibitors of the MDM2-p53 protein-protein interaction to reactivate p53 function: A novel approach for cancer therapy. Annu. Rev. Pharmacol. Toxicol..

[180] Dickens M.P., Fitzgerald R., Fischer P.M. (2010). Small-molecule inhibitors of MDM2 as new anticancer therapeutics. Semin. Cancer Biol..

[181] Azmi A.S., Philip P.A., Aboukameel A., Wang Z., Banerjee S., Zafar S.F., Goustin A.S., Almhanna K., Yang D., Sarkar F.H. (2010). Reactivation of p53 by novel MDM2 inhibitors: Implications for pancreatic cancer therapy. Curr. Cancer Drug Targets.

[182] Gu L., Zhang H., Liu T., Zhou S., Du Y., Xiong J., Yi S., Qu C.K., Fu H., Zhou M. (2016). Discovery of dual inhibitors of MDM2 and xiap for cancer treatment. Cancer Cell.

[183] Vasilena Z., Minglei B., Justin J.O., Monicah N., Bruce E.K., Amy H.T. (2018). Inhibition of Well-Established Pancreatic and Triple Negative Breast Tumor Growth by Blocking the Most Downstream Signaling Module, SIAH, in the Oncogenic ERBB/K-RAS Signaling Pathway.

[184] Qi J., Nakayama K., Gaitonde S., Goydos J.S., Krajewski S., Eroshkin A., Bar-Sagi D., Bowtell D., Ronai Z. (2008). The ubiquitin ligase SIAH2 regulates tumorigenesis and metastasis by HIF-dependent and -independent pathways. Proc. Natl. Acad. Sci. USA.

